# Supplementary material for: Causal Relationship between Aging and Anorexia Nervosa: A White-Matter-Microstructure-Mediated Mendelian Randomization Analysis
Source: Biomedicines. 2024 Aug 16;12(8):1874. doi: 10.3390/biomedicines12081874 (PMC11351342; doi:10.3390/biomedicines12081874)
Supplement: Supplementary file 1 [file biomedicines-12-01874-s001.zip › Supplementary Figure/Figure S8/Leaveoneout plot of MR analysis for TL to ubm-b-1516.pdf]

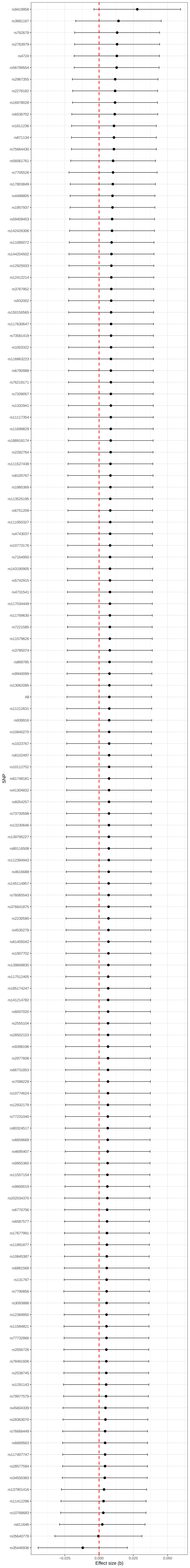
rs9419958
rs3891167
rs762679
rs2763979
rs4724
rs56799554
rs2967355
rs2276182
rs16978028
rs6536702
rs1611236
rs871134
rs75664430
rs56061761
rs7705526
rs17803849
rs4498805
rs1957937
rs59409453
rs142426306
rs11085072
rs144204502
rs12925933
rs12412214
rs3767952
rs932002
rs150150565
rs117630647
rs73581419
rs1003322
rs116863223
rs6790988
rs76219171
rs7209057
rs1332941
rs11117354
rs11699829
rs188918174
rs2282764
rs111527438
rs8105767
rs1985369
rs113525195
rs6751209
rs111950327
rs4743037
rs10773176
rs7164950
rs143190905
rs5742915
rs4731541
rs117034449
rs11769630
rs7221585
rs11579626
rs3785074
rs869785
rs9940099
rs13062095
A8
rs11212631
rs939916
rs10840270
rs1023767
rs8102497
rs10112752
rs61748181
rs41304832
rs6054257
rs73730598
rs13230646
rs139795227
rs80116508
rs112394943
rs4616688
rs145114957
rs76065543
rs376641875
rs2230590
rs4530278
rs61405042
rs1907702
rs139669835
rs117512405
rs185174247
rs141214782
rs6007020
rs2555104
rs28502153
rs9398196
rs2977608
rs66731853
rs7099229
rs10774624
rs12932179
rs77231040
rs80324517
rs6659669
rs4695407
rs9955360
rs11557154
rs9600019
rs202034370
rs6776756
rs6587577
rs17677991
rs11991877
rs10645387
rs6881568
rs131797
rs7790856
rs3093888
rs12369950
rs11584821
rs77732866
rs2056726
rs78491606
rs2538745
rs1291143
rs79977579
rs45604339
rs28363070
rs76666449
rs6669563
rs117407747
rs28577594
rs34550383
rs137901416
rs11412296
rs10768683
rs611646
rs35640778
rs35446936
SNP
-0.025
0.000
0.025
0.050
Effect size (b)
